# A Practical Treatise on the Diseases of Women

**Published:** 1869-05

**Authors:** 


A Practical Treatise on the Diseases of Women. By T. GAILLARD THOMAS, M.D., Professor of Obstetrics and Diseases of Women and Children, in the College of Physicians and Surgeons, New York; Physician to the Bellvue Hospital; etc., etc., etc. With 225 Illustrations. Second Edition, revised and improved. Philadelphia: Henry C. Lea. 1869. 627 pages. For sale by S. C. Griggs & Co.

It is but a few months since we noticed and commended to our readers the first edition of this work. This edition is published in excellent style, and has been carefully revised by the author. It is an excellent practical treatise on the diseases of the non-pregnant female.

